# Genome Sequence of the Endosymbiont *Rickettsia peacockii* and Comparison with Virulent *Rickettsia rickettsii*: Identification of Virulence Factors

**DOI:** 10.1371/journal.pone.0008361

**Published:** 2009-12-21

**Authors:** Roderick F. Felsheim, Timothy J. Kurtti, Ulrike G. Munderloh

**Affiliations:** Department of Entomology, University of Minnesota, Saint Paul, Minnesota, United States of America; University of Hyderabad, India

## Abstract

*Rickettsia peacockii,* also known as the East Side Agent, is a non-pathogenic obligate intracellular bacterium found as an endosymbiont in *Dermacentor andersoni* ticks in the western USA and Canada. Its presence in ticks is correlated with reduced prevalence of *Rickettsia rickettsii*, the agent of Rocky Mountain Spotted Fever. It has been proposed that a virulent SFG rickettsia underwent changes to become the East Side Agent. We determined the genome sequence of *R. peacockii* and provide a comparison to a closely related virulent *R. rickettsii*. The presence of 42 chromosomal copies of the ISRpe1 transposon in the genome of *R. peacockii* is associated with a lack of synteny with the genome of *R. rickettsii* and numerous deletions via recombination between transposon copies. The plasmid contains a number of genes from distantly related organisms, such as part of the glycosylation island of *Pseudomonas aeruginosa*. Genes deleted or mutated in *R. peacockii* which may relate to loss of virulence include those coding for an ankyrin repeat containing protein, *DsbA*, *RickA*, protease II, *OmpA*, *ScaI*, and a putative phosphoethanolamine transferase. The gene coding for the ankyrin repeat containing protein is especially implicated as it is mutated in *R. rickettsii* strain Iowa, which has attenuated virulence. Presence of numerous copies of the ISRpe1 transposon, likely acquired by lateral transfer from a *Cardinium* species, are associated with extensive genomic reorganization and deletions. The deletion and mutation of genes possibly involved in loss of virulence have been identified by this genomic comparison. It also illustrates that the introduction of a transposon into the genome can have varied effects; either correlating with an increase in pathogenicity as in *Francisella tularensis* or a loss of pathogenicity as in *R. peacockii* and the recombination enabled by multiple transposon copies can cause significant deletions in some genomes while not in others.

## Introduction

*Rickettsia peacockii* is an obligate intracellular bacterium identified in Rocky Mountain wood ticks (*Dermacentor andersoni*) from Montana, USA [1]. It is of interest to rickettsiologists due to its co-localization on the eastern side of the Bitterroot Valley with a much reduced prevalence of *D. andersoni* infected with *Rickettsia rickettsii*, while spotted fever ravaged the west side of the valley [2] [3]. Thus began the study of a phenomenon considered as evidence for interference, where the presence of *R. peacockii* in *D. andersoni* ticks may prevent the transovarial transmission of *R. rickettsii* and therefore limit its spread in the tick population. It is not clear whether this interference is an active process or simply a case in which ticks carrying *R. peacockii* have a reproductive advantage because they do not suffer the reduced fecundity associated with *R. rickettsii* infection [4]. Surveys of rickettsiae in tick populations around the western US and Canada have shown that *R. peacockii* is widespread in *Dermacentor* ticks and *R. rickettsii* is relatively rare [1], [5]. While *R. peacockii* is closely related to *R. rickettsii*, it is not a pathogen of mammals and not deleterious to ticks.

The purpose of this work was to determine the sequence of the *R. peacockii* genome and compare it to the genome of it's nearest pathogen relative, *R. rickettsii*. Although the *R. peacockii* genome is similar in size (1.29Mb) to those of other spotted fever group (SFG) rickettsiae and there is high homology between many of their genes, the genome of *R. peacockii* has several gene deletions and mutations that may account for its lack of pathogenicity. In addition to the genome sequence of the Iowa strain of *R. rickettsii* [6] it represents a valuable set of data for comparison with the genomes of related pathogenic rickettsiae. The most dramatic difference between the genomes of *R. peacockii* and *R. rickettsii* is the presence of the ISRpe1 transposon and the effects multiple transposon copies have had as a point of homology for recombination, resulting in numerous deletions and genome shuffling (this manuscript). In contrast, genome comparisons of virulent and non-virulent *Francisella tularensis* showed that transposon mediated recombination and shuffling of gene order occurred in the pathogenic strains rather than the non-pathogenic strain [7]. *R. peacockii* is also among the growing list of rickettsiae harboring plasmids which are apparently lacking in *R. rickettsii*.

## Results and Discussion

*R. peacockii* accession numbers, **chromosome** GenBank:**CP001227**,**plasmid** GenBank:**CP001228.**

The genome sequence of *R. peacockii* strain Rustic was determined; the size of the circular chromosome (RPR) is 1,288,492 bp and the size of the circular plasmid (pRPR) is 26,406 bp. The gene sequences of *R. peacockii* were found to be most similar to those of virulent *R. rickettsii* Sheila Smith (SS) and avirulent *R. rickettsii* Iowa. The genome of the non-pathogen *R. peacockii* was compared to the genome of its closest pathogenic neighbor *R. rickettsii* SS in order to identify differences that may relate to pathogenicity. Presence of laterally transferred DNA in the genome of *R. peacockii* is the most striking difference between them; including a plasmid, the ISRpe1 transposon and three chromosomal regions of *Rickettsia bellii*-like DNA containing Tra genes. The locations of chromosomal DNA sequences present in *R. peacockii* and lacking in *R. rickettsii* SS are shown in Text S1.

### Impact of ISRpe1 Transposons on the *R. peacockii* Genome

There are 40 copies of the transposon and 2 transposon fragments on the chromosome and 2 copies of the transposon on the plasmid. Most ISRpe1 transposons contain an intact transposase coding sequence (31 of 42) while 11 contain frameshift mutations or internal stop codons. There are no other types of transposases annotated as genes on the chromosome and only three other types of transposon pseudogenes are found on the chromosome, two of which are also found in *R. rickettsii* and one within a fragment of the tra cluster.

**Figure 1:**
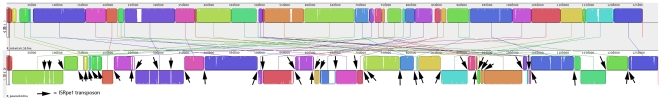
Alignment of *Rickettsia rickettsii* and *Rickettsia peacockii* genomes. The alignment of the *Rickettsia rickettsii* SS and *Rickettsia peacockii* genomes using progressive Mauve with default parameters shows the lack of synteny between the genomes of these closely related organisms. The breakpoints of the syntenic blocks in *R. peacockii* are largely associated (31 of 37) with the ISRpe1 transposon, indicated with black arrows. The genome on top is that of *R. rickettsii* SS (reference genome) and that below is *R. peacockii*.

Recombination between the transposons has resulted in a dramatic shuffling of gene order between the *R. peacockii* and *R. rickettsii* genomes (Figure 1). A dot plot comparison of the two genomes is shown in Figure S1. By comparing the two genomes using Mauve [8] we found that an ISRpe1 transposon in *R. peacockii* co-localized to 31 of 37 junctions between syntenic blocks. Numerous deletions co-localized to copies of the transposon as well, suggesting the deletions occurred during this recombination. To determine the extent of correlation between deletions and the presence of transposons, the backbone file from the Mauve comparison, Artemis [9] and blastn was used to locate copies of ISRpe1 within 5 bp of the deletion junctions. All deletions over 100 bp in size were examined. There are transposons not associated with gdeletions or changes to synteny, transposons that locate to changes in synteny with or without deletion, and transposons that locate to a point of deletion but do not affect synteny. Deletions of the latter variety likely occurred when two transposons integrated near each other followed by recombination between them and deletion of the intervening DNA. Text S2 shows that for all deletions greater than 100 bp in size, 71.4% of deletions (25 out of 35) are flanked by one or two ISRpe1 transposons. There are also 3 smaller deletions flanked by transposons. It is possible that some of the small deletions resulting in frameshift mutations or split genes are the result of inexact DNA repair following excision of the transposon as it moved to a new location. In contrast to this study, genome comparisons of virulent and non-virulent *Francisella tularensis* showed that transposon mediated recombination and shuffling of gene order occurred in the pathogenic strains rather than the non-pathogenic strain [7], also they did not find deletions associated with transposon mediated recombination as seen in *R. peacockii*.

**Figure 2:**
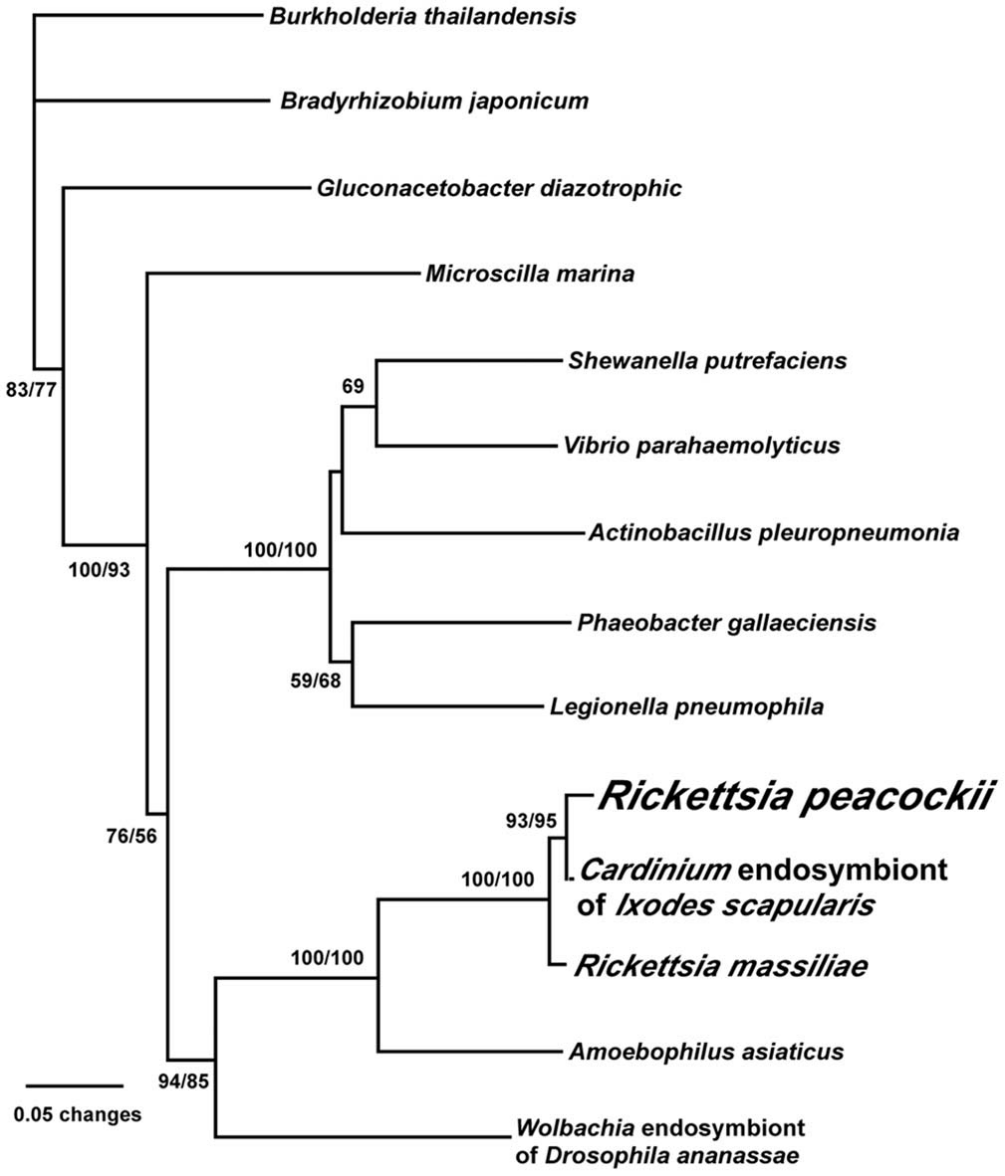
Phylogenetic analysis of ISRpe1 transposon. Neighbor joining (NJ) and maximum parsimony (MP) analyses included 14 taxa. Exclusion of gaps left 309 amino acids for the analyses; 105 amino acids were constant, 36 of the variable amino acids were parsimony uninformative and 168 of the variable amino acids were parsimony informative. Bootstrap analysis involved 2,000 replicates: top number is NJ bootstrap value and bottom number the MP bootstrap value. Genbank references for the proteins found in Text S7.

The transposon ISRpe1, originally identified in *R. peacockii*, [10] is also found twice in the *R. massiliae* genome along with a gene fragment (E = 0.0; RMA_0538 and RMA_0748) and a frameshifted mutant copy is found on the *R. felis* plasmid (E = 4e−154; RF_p48). The genomic locations for the copies of ISRpe1 in *R. massiliae* are different from those found in *R. peacockii*, indicating the transposition events occurred independently rather than in a common ancestor. Homologs of ISRpe1 are found three times (E = 5e−173; Aasi_0934, Aasi_0956 and Aasi_0884) in the genome of *Candidatus* Amoebophilus asiaticus, a member of the phylum *Cytophaga-Flavobacterium-Bacteroides* (CFB) and endosymbiont of *Acanthamoeba* [11]. The phylum also contains *Candidatus* Cardinium endosymbionts of arthropods. *C*. Cardinium spp. are related to *C.* A. asiaticus and closely related to one another, yet exist in a wide range of arthropod species [12]. This is indicative of horizontal transmission and could put these bacteria in contact with various species of rickettsiae. A phylogenetic tree, Figure 2, shows the close relationship between ISRpe1 and the *Candidatus* A. asiaticus transposon, but given the presence of this transposon in few rickettsiae, we suggest the transposon was transferred to these few rickettsiae in the recent past rather than from *C*. A. asiaticus. A possible source of the transposon is a *C.* Cardinium species from *D. andersoni* ticks that is similar to an endosymbiont cultured from *Ixodes scapularis* ticks [13]. To explore this link, genomic DNA from the cultured *C*. Cardinium spp. was used as template in a PCR reaction with ISRpe1 primers not previously used in the lab and the sequence of the product was determined. The DNA sequence of the 931 bp *C*. Cardinium PCR product shares 98% identity with the most homologous ISRpe1 copy in *R. peacockii.* The derived amino acid sequence was added to the phylogenetic tree shown in Figure 2. These results support the hypothesis that the transposon was transferred from a *C*. Cardinium species to a recent ancestor of *R. peacockii*.

### Tra Gene Cluster of *R. peacockii*

The three regions of *R. bellii*-like DNA in the chromosome are located at nucleotides 142335–149261 (one end of the tra cluster with genes *TraB*, *TraE*, leucine-rich protein gene and a *TraV* fragment), 806589–813116 (with degraded genes for a permease, *TraA* and *TraD*) and 497519–499744 (other end of the tra cluster with U gene). The phenomenon of lateral transfer of the tra cluster was first observed in the *R. massiliae* genome [14]. An ISRpe1 transposon is present at four of the six junctions of these three regions and the remaining two junctions are the tRNAVal gene and a chimeric tRNA gene. It was difficult to determine the junction at 149261 (the chimeric tRNA gene) as this area has been lost in *R. rickettsii.* This junction border was chosen due to blastn comparison with *R. conorii* that shows the highest homology (98%) upstream from nucleotide 149262 and no homology downstream. This correlates well with events of integration at tRNA genes by integrons such as the tra cluster [15]. The leucine-rich protein gene (RPR_00830) in the TraBE region is not found in *R. massiliae* and only shows homology (97%) to RBE_0439 of *R. bellii* which is near *TraE,* suggesting either strong selection for this gene sequence uniquely in *R. peacockii* and *R. bellii* or independent introduction of the tra cluster within the rickettsiae. Also, the permease pseudogene in the TraAD region of *R. peacockii* is not found in *R. massiliae*, but found in the tra cluster of *R. canadensis* (A1E_02610 and A1E_02615). In *R. peacockii* it appears the tra cluster integration preceded the arrival of the ISRpe1 transposon which then transposed into the tra cluster and split it into the three remaining regions by recombination and presumable deletion of the bulk of the tra cluster. The genome of *R. rickettsii* SS does not contain this tra cluster but may have a remnant 227 bp fragment located at nucleotides 7243640–724132 near the tRNAVal gene [14]. When this 227 bp region from *R. peacockii* is compared with other rickettsiae using blastn, *R. rickettsii* shows 68% identity (E = 6e−14) while *R. bellii* shows 88% identity (E = 1e−73) and *R. massiliae* shows 85% identity (E = 4e−67). If the 227 bp region of *R. rickettsii* shared ancestry with the tra cluster of closely related *R. peacockii*, one would expect the percent identity to *R. rickettsii* to be higher than that of *R. bellii* and *R. massiliae*.

### Features of *R. peacockii* Plasmid

**Figure 3:**
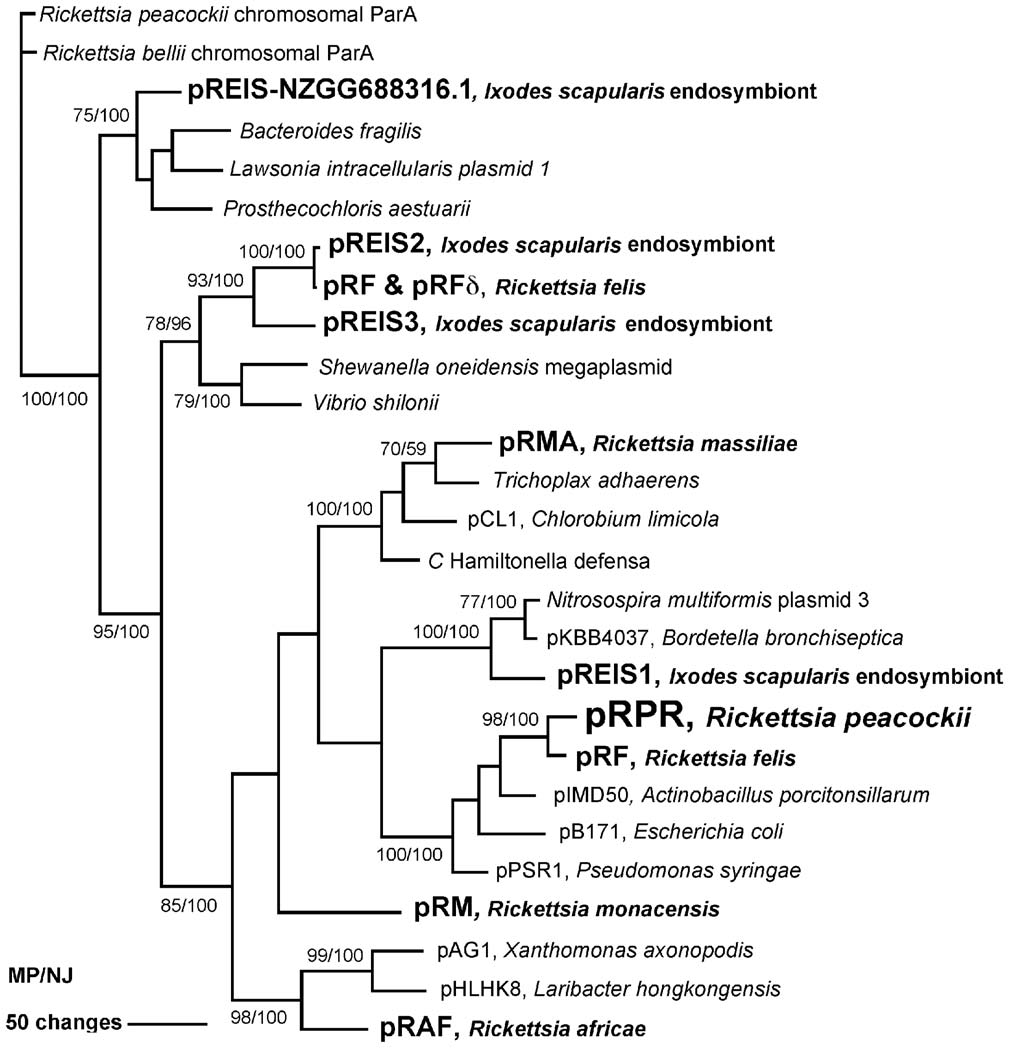
Maximum parsimony analysis of 27 parA proteins. Exclusion of gaps left 166 amino acids for the analysis; 8 amino acids were constant, 2 of the variable amino acids were parsimony uninformative and 156 of the variable amino acids were parsimony informative. Bootstrap analysis involved 1,000 replicates: numbers at selected branches are the NJ (MP/NJ) bootstrap values that were ≥50%. The top two or three blastP hits to rickettsial plasmid parA's with E = >1e−30 were chosen for the analysis. The parA from the *Trichoplax adhaerens* genome project is likely from a bacterium associated with this simplest of eukaryotes as several contigs have homology to Rickettsiales. The parA proteins from the rickettsial endosymbiont of Ixodes scapularis (REIS) were added to the analysis following PCR and sequencing to confirm the presence of the genes in our REIS isolate (Baldridge et. al., in preparation). Maximum parsimony and neighbor joining (not shown) phylograms were congruent. Genbank references for the proteins found in Text S7.

The 26 kb plasmid of *R. peacockii* (pRPR) contains 20 putative genes (Table 1), two of which are involved in plasmid maintenance and replication, *ParA* and *DnaA*. The *ParA* gene RPR_p01 and the two neighboring genes RPR_p02 and RPR_p03 are most closely related to RF_p23, RF_p22 and RF_p21 of *R. felis* and flanked by 56 bp inverted repeats, whereas the *R. massiliae* plasmid has an unrelated *ParA* gene. A phylogenetic tree was made comparing rickettsial plasmid borne parA proteins with their closest blast hits (Figure 3). It is interesting that the *ParA* genes on rickettsial plasmids fall into diverse groups suggesting foreign plasmids have periodically entered rickettsiae. ParA is the likely determinant of compatibility so entrance of a new parA gene enables a second plasmid to be maintained. The C-terminal domain of *DnaA* is similar to the plasmid-borne DnaA-like proteins of *R. massiliae* RMA_p01, *R. felis* RF_p05 and also the smaller version in *R. felis* RF_p19. The N-terminal domain is similar to the DnaA-like protein of *R. monacensis*. The plasmid contains five genes, RPR_p06 – RPR_p10, most closely related to orfs B, C, D, F, G found in a region termed the glycosylation island in *Pseudomonas aeruginosa*, shown to be involved (orfs A, N and E) in flagellar glycosylation [16]. The gene order is mostly maintained in this gene cluster on pRPR with only orf E deleted and orfB flipped between the repeats shown in the annotation. The GC content of this region is 48.4% vs. 34.7% for the entire plasmid and 32.6% for the *R. peacockii* chromosome, another indication beyond the homology for lateral transfer. The function of these five genes in *R. peacockii* is unknown but by homology they appear to be involved in phospholipid biosynthesis and may be maintained to increase the flow of glycerol-3-phosphate into the phospholipid biosynthesis pathway given that *R. peacockii* has a frameshift mutation in the glycerol-3-phosphate dehydrogenase gene (Table 2, location 96554..97530). This gene is necessary to make glycerol-3-phosphate from dihydroxyacetone phosphate (DHAP) in rickettsiae. Due to the absence of the glycolytic pathway, rickettsiae are unable to synthesize DHAP from fructose-1,6-diphosphate and must import it from the host cell [17]. Rickettsiae commonly have a glycerol-3-phosphate transporter as well as a DHAP transporter so can obtain glycerol-3-phosphate from the host cell directly or indirectly, while only *R. peacockii* has a mutant copy of the glycerol-3-phosphate dehydrogenase gene, so is dependent on import of glycerol-3-phosphate alone. This mutation in *R. peacockii* may limit the amount of glycerol-3-phosphate available for phospholipid biosynthesis and the presence of these 5 genes on the plasmid may alleviate this problem.

**Table 1:** Comparison of plasmid sequence to Genbank using blastP, or blastX for frameshifts and fragments.

Location on pRPR	Top blast hit
RPR_p01	*Rickettsia felis* RF_p23 Plasmid stability protein ParA2, E = 2e−92, Positives = 194/211 (91%) Not found in other rickettsiae.
RPR_p02	*Rickettsia felis* RF_p22 Hypothetical protein, E = 3e−25, Positives = 69/82 (84%)
RPR_p03	*Rickettsia felis* RF_p21 Hypothetical protein, E = 3e−29, Positives = 90/130 (69%)
RPR_p04	*Rickettsia massiliae* RMA_p01 DnaA-like replication initiator protein E = 2e−123, Positives = 435/798 (54%)
5154..6508	*Rickettsia bellii* RBE_0057 Type I restriction-modification system methyltransferase subunit, pseudogene E = 0.0, Positives = 392/450 (87%)
RPR_p05	*Rickettsia massiliae* RMA_0538 Transposase, E = 0.0, Positives = 360/364 (98%)
RPR_p06	*Pseudomonas aeruginosa* PA2G_00065 UDP-3-O-[3-hydroxymyristoyl] glucosamine N-acyltransferase, E = 6e-72, Positives = 165/205 (80%)
RPR_p07	*Pseudomonas aeruginosa* PA2G_00064 Phenylpropionate dioxygenase and related ring-hydroxylating dioxygenases, E = 3e−164, Positives = 315/375 (84%)
RPR_p08	*Pseudomonas aeruginosa* PaerPA_01001561 (PA2G_00062) FabG, 3-ketoacyl-(acyl-carrier-protein) reductase, E = 6e−101, Positives = 221/255 (86%)
RPR_p09	*Pseudomonas aeruginosa* PA2G_00061 FabH, 3-oxoacyl-[acyl-carrier-protein], E = 3e−158, Positives = 291/319 (91%)
RPR_p10	*Pseudomonas aeruginosa* PA2G_00060 Acyl carrier protein, E = 1e−25, Positives = 65/72 (90%)
RPR_p11	*Candidatus* Amoebophilus asiaticus Aasi_0982 ABC-type transport system, ATPase and permease components, E = 0.0, Positives = 442/575 (76%)
14357..15476	*Rickettsia massiliae* RMA_0746 Transposase, pseudogene E = 3e−140, Positives = 257/272 (94%) Not found in other rickettsiae.
RPR_p12	*Rickettsia monacensis* RM-p7 Hsp1, Molecular chaperone (small heat shock protein), E = 1e−56, Positives = 139/176 (78%)
RPR_p13	*Rickettsia monacensis* RM-p6 Hsp2, Molecular chaperone (small heat shock protein), E = 1e−73, Positives = 146/154 (94%)
RPR_p14	*Orientia tsutsugamushi* OTT_1892 Transposase-like, E = 5e−104, Positives = 241/303 (79%)
RPR_p15	*Rickettsia massiliae* RMA_0538 Transposase, E = 0.0, Positives = 358/364 (98%)
19828..20073	*Wolbachia* WD_0253 Transposase fragment, E = 1e−27, Positives = 66/77 (85%)
20088..21168	*Rickettsia felis* RF_p41 Transposase, pseudogene E = 3e−164, Positives = 330/356 (93%)
RPR_p16	*Erwinia tasmaniensis* ETA_pET460420 Putative lipoprotein, E = 2e−13, Positives = 67/121 (55%)
RPR_p17	*Rickettsia bellii* RBE_0152 SMR-type multi-drug efflux transporter, E = 2e−41, Positives = 94/103 (91%) Not found in other Rickettsiae.
RPR_p18	*Methanosarcina barkeri* Mbar_A2604 TPR repeat-containing protein, E = 8e−13, Positives = 64/116 (55%)
RPR_p19	First half of protein is fragment of *Rickettsia conorii* protein PS 120 (Sca4) E = 2e−68, Positives = 164/223 (73%). Second half of protein yields no homologies. Apparent chimeric protein.
RPR_p20	*Rickettsia akari* A1C_03990 hypothetical protein, partial homology, E = 8e−92, Positives = 228/323 (70%)

**Table 2:** Nonsense mutations and split genes in *R. peacockii* relative to *R. rickettsii* Sheila Smith.

Location of gene in *R. peacockii*	Gene and type of mutation
10935..10988 and 151409..151545	Small hypothetical gene A1G_03990 split by recombination
**15676..16998**	**Methyltransferase A1G_03950, premature stop codon**
around 188490	Recombination near this point (219340 in SS) split hypothetical gene A1G_01175
29106..29608	BioY family protein RrIowa_0811 (not annotated in SS), frameshift
**96554..97530**	**NAD(P)H-dependent glycerol-3-phosphate dehydrogenase A1G_03470, frameshift**
186175..186749	Cytochrome oxidase biogenesis protein A1G_00265, frameshift
298139..299185	AFG1-like ATPase A1G_01615, premature stop codon
441508..442904	NAD/NADP transhydrogenase beta subunit A1G_00635, N-terminal truncation of 82 AA's due to frameshift.
462858..463848	SAM-dependent methyltransferase domain A1G_00720, frameshifts, results in lack of TPR domain in RPR_02715
559562..560115	(di)nucleoside polyphosphate hydrolase A1G_06270, frameshift and internal stop codon
617196..618666	Patatin b1 precursor A1G_05085, frameshift, C-terminal truncation but contains the complete patatin superfamily domain.
630337..631282	Hypothetical protein (conserved rickettsial) A1G_04725, frameshift
884549..885426	Acyltransferase COG1835 A1G_07015 (likely pseudogene), frameshift
**888608..892941**	**OmpA A1G_06990, three frameshifts**
917962..918565	Ankyrin repeat-containing protein A1G_04305, frameshift
998131..999374	AmpG A1G_03035, frameshift
1007548..1009092	Cytochrome c oxidase, subunit I, A1G_02985, frameshifts
1139934..1140955	Hypothetical protein A1G_02790, two frameshifts
1171010..1172275	Conserved hypothetical protein A1G_02605, two frameshifts
**1176591..1178159**	**Putative phosphoethanolamine transferase A1G_02570, two frameshifts**

Also found on the plasmid are two small heat shock genes, one (RPR_p13) appears to be a common feature present on rickettsial plasmids [18]. RPR_p13 homologs are not represented on the chromosomes of other rickettsiae except *R. felis* (RF_1004) but this is an unusual case in that the other *R. felis* chromosomal copy (RF_1005) has a frameshift mutation and RF_1004 may have arisen via recombination with the plasmid copy. RPR_p12 is more similar to small heat shock proteins found on the chromosome of all rickettsiae but phylogenetic analysis shows this plasmid copy falls into a third group of rickettsial small heat shock proteins (Text S3). A comparison of the three small heat shock proteins of *R. peacockii* (including chromosomal copy RPR_2300) using Kyte-Doolittle plots shows the degree of N-terminal hydrophobicity varies from high to low between the three proteins with the two plasmid copies having the greatest difference (Text S4). In yeast the strength of this N-terminal hydrophobicity determines the strength of interaction of these chaperones with their target proteins [19], [20]. A family of these chaperones with a range of strengths of interaction may well help rickettsiae survive changing environmental temperatures during their life cycles in arthropods. It is also possible that individual target proteins or membranes benefit from a specialized chaperone. The plasmid contains genes for two transporters; RPR_p11 is an ABC type with ATPase and permease domains with strong homology (E = 0.0) to Aasi_0982 from *Candidatus* A. asiaticus and no homology to known rickettsial genes. The second transporter RPR_p17 is an SMR-type multi-drug efflux transporter and the only other rickettsia to have a homolog of RPR_p17 is *R. bellii*, while next closest relatives are in CFB group of bacteria. Other genes on the plasmid code for various transposases, a putative lipoprotein, a TPR repeat-containing protein and an apparent chimeric protein. All the plasmid genes with rickettsial chromosomal homologs have far lower homology to those of *R. peacockii* or *R. rickettsii* and higher homology to other more distantly related rickettsiae, which is an indication of horizontal gene transfer to the plasmid, as seen in pRF of *R. felis* [21]. The exceptions are the two ISRpe1 transposon copies on the plasmid.

### Deletions and Mutations in *R. peacockii* vs. *R. rickettsii* Strain Sheila Smith

Deletions in *R. peacockii* vs *R. rickettsii* SS are shown in Table 3. Deletions greater than 100 bases were examined as well as smaller deletions that disrupted genes or were within 5 bases of ISRpe1 transposons. Nonsense mutations resulting from premature stop codons and small deletions or insertions causing frameshifts in *R. peacockii* vs *R. rickettsii* SS are shown in Table 2. It appears that some deletions and mutations in *R. peacockii* may be responsible for its lack of pathogenicity and are focused upon in this section. Possible candidate genes include those coding for an ankyrin repeat containing protein, *DsbA*, *RickA*, Protease II, *OmpA*, *Sca1*, and a putative phosphoethanolamine transferase. The deletion located at SS coordinates 869412..871928 (Table 3) was likely deleted in *R. peacockii* during recombination between ISRpe1 transposons and contains a gene coding for one of the two larger ankyrin repeat containing proteins in *R. rickettsii* SS (A1G_05165). Ankyrin repeat proteins have been shown to be effector proteins or virulence factors in several pathogens [22]. In another member of the order Rickettsiales, AnkA is rapidly translocated to the host cell and phosphorylated by host cell kinases upon *Anaplasma phagocytophilum* binding to the host cell [23]. AnkA also binds DNA and alters transcription of defense related genes in HL-60 cells infected with *A. phagocytophilum* or transfected with an AnkA expression plasmid [24]. Transcript levels for ankA (APH_0740) were shown to be 2.3 to 3 fold higher in *A. phagocytophilum* grown in mammalian cells versus tick cells [25] and in *R. rickettsii* transcript levels of A1G_05165 were 3.2 fold higher in mammalian cells versus tick cells as well as being one of few differentially expressed genes detected [26]. Moreover, this gene that is deleted in *R. peacockii* is mutated in *R. rickettsii* Iowa, which has attenuated virulence compared to *R. rickettsii* SS [6]. The deletion in the Iowa gene (RrIowa_1113) removes three of the four ankyrin repeats from the protein. Strengthening the case for this as a virulence factor is the observation that this gene has been deleted in *Rickettsia monacensis* (R. Felsheim, unpublished) and is not found in *R. bellii*, both non-pathogenic for humans. Recently the putative genome sequence from the non-pathogenic rickettsial endosymbiont of *Ixodes scapularis* (REIS) has been released and this ank gene also appears to have been deleted in a similar manner to that seen in *R. monacensis*. A remnant of the gene found at nucleotides 53520–53589 of REIS contig ACLC01000066.1 are homologous to the 3′ end of the ank gene. There is about a 3.7 kb deletion in this region of the REIS genome compared to *R. rickettsii*. All of the rickettsial pathogens for which genome sequence is available have this gene, except *Rickettsia akari.*

**Table 3:** Deletions in *R. peacockii* relative to *R. rickettsii* Sheila Smith.

Location in *R. rickettsii* SS	Size of deletion	Genes or gene products contained in deletion
14498..15082	583	A1G_00085 truncated, antitoxin A1G_00090 lost and A1G_00095 truncated
**19653..25701**	**6047**	**A1G_00130, Cell surface antigen Sca1-like**
36902..37126	223	A1G_00215, Part of dihydrofolate reductase gene
48049..52257	4207	Region of gene fragments resulting from gene reduction
219917..220001	83	A1G_01175, 3′ end of hypothetical gene, also split by rearrangement at bp 219337
231815..231898	82	A1G_01245, radical SAM family enzyme gene, results in frameshift
232280..232351	70	A1G_01245, 3′ end of radical SAM family enzyme gene
258009..266392	8382	Gene fragments for penicillin binding protein and fragments of Sca8 resulting from gene reduction
333287..333616	328	A1G_01880, hypothetical gene
372621..372708	86	Small intergenic deletion
**381886..381961**	**74**	**A1G_02165, Protease II, results in frameshift**
444166..444318	151	A1G_02530, small hypothetical gene
**494539..498554**	**4014**	**A1G_02820, 02825, 02830, ABC transporter component genes**
506267..506762	494	Gene fragments resulting from gene reduction
561101..563976	2874	Gene fragments of RND efflux transporter gene
**588183..589234**	**1050**	**A1G_03355, Protein-disulfide isomerase, DsbA gene**
614711..615355	643	Missing part of A1G_03530 at point of transposon insertion
619349..619541	191	Small intergenic deletion
687771..689062	1290	Gene fragments resulting from gene reduction
691605..692087	481	A1G_04035 hypothetical gene
708170..708343	172	Small intergenic deletion
715158..716455	1296	A1G_04170 hypothetical gene
723952..728058	4105	Region of gene fragments resulting from gene reduction
730884..731135	250	A1G_4290, Gene fragment
741167..741466	298	A1G_4355, Gene fragment
742387..742859	471	A1G_4365, Gene fragment
747947..753101	5153	Region of gene fragments resulting from gene reduction
**780788..783183**	**2394**	**A1G_04605, YhbC and A1G_04620, transcriptional regulator rirA and gene fragments resulting from gene reduction**
787638..788105	466	A1G_04660 truncated (S4 ribosomal and related proteins)
807386..807708	321	A1G_04775 hypothetical
813412..813703	290	Gene fragments resulting from gene reduction
839333..839525	191	A1G_04970 truncated, hypothetical gene
**841975..846686**	**4710**	**A1G_05015 RickA gene, A1G_04995 to A1G_05010 Succinyl-CoA:3-ketoacid-coenzyme A transferase subunits A and B and gene fragments**
856816..859634	2817	Gene fragments resulting from gene reduction
**869412..871928**	**2515**	**A1G_05165 ankyrin repeat protein gene, also mutated in ** ***R. rickettsii*** ** Iowa (RrIowa_1113)**
913278..917419	4140	Region of gene fragments resulting from gene reductions
975118..975936	817	A1G_05855, two copies of LPS biosynthesis protein gene recombined into one
1000150..1000436	285	Gene fragments resulting from gene reduction
1139429..1140228	798	Gene fragments of COG2602 Beta-lactamase class D
1145752..1146794	1041	Gene fragment resulting from gene reduction

While most bacteria have a single *DsbA* gene, *R. rickettsii* SS and other rickettsiae have two *DsbA* genes, one (A1G_03355) is deleted in *R. peacockii* due to recombination between two ISRpe1 transposons (Table 3, location 588183..589234). *DsbA* codes for a protein-disulfide oxidoreductase which catalyzes disulfide-bond formation in the periplasm during the folding of secreted proteins. Both rickettsial DsbA proteins are predicted to be anchored into the membrane, one via a transmembrane domain and one as a lipoprotein. In pathogenic *Vibrio cholerae*, the DsbA homolog (TcpG) is responsible for the folding, maturation and secretion of virulence factors [27]. The importance of DsbA for virulence has been demonstrated in a variety of organisms [28], [29], [30], [31], [32], [33], [34]. Compensation for this deletion by the other copy of DsbA is likely for some functions, but in *Neisseria meningitides* which has three DsbA homologs, they vary in functional activity in complementation assays [35].

*RickA* was previously shown to be truncated by the ISRpe1 transposon in *R*. *peacockii* and interaction of *R. peacockii* with actin was found to be lacking [10]. We show that most of the *RickA* gene has been deleted along with the neighboring succinyl-CoA:3-ketoacid-coenzyme A transferase genes during recombination between transposons (Table 3, location 841975..846686). Actin based motility is thought to mediate intracellular and cell-to-cell movement of rickettsiae. Time-lapse photography of our GFP expressing *R. peacockii* shows them to be non-motile compared to other rickettsiae observed (unpublished data). The protease II gene in *R. peacockii* (Table 3, location 381886..381961) has a 74 bp deletion causing a frameshift in the middle of the gene. Proteases have been shown to be important for binding and entry in a wide variety of organisms and protease II is an S9A type protease, the type shown to be necessary for entry of *Trypanosoma cruzi* into host cells [36]. The three frameshift mutations in the *OmpA* gene of *R. peacockii* have previously been discussed [37]. In the avirulent strain *R. rickettsia* Iowa, *OmpA* is also truncated as a result of a frameshift mutation [6] that differs from those in the *R. peacockii OmpA* gene. *Sca1*, which is in the same superfamily of autotransported surface proteins as *OmpA*, is deleted in *R. peacockii* via transposon insertion upstream of the *Sca1* gene and also near the stop codon (Table 3, location 19653..25701), followed by recombination and deletion of a 6kb DNA fragment containing the *Sca1* gene. *Sca1* is present in all other *Rickettsia* spp. and the type of selection pressure on the N-terminal passenger domain implicates the N-terminal domain of this autotransported protein in interactions with the host cell [38]. Outer membrane proteins implicated in binding of rickettsiae to host cells, include OmpA [39], OmpB [40], RP828, RC1281, and the autotransporter domain of OmpB [41]. Of these only OmpA is defective in *R. peacockii.* It is possible that rickettsiae use different members of this Omp family to bind to different host cell types or cells of different species.

Since the N-terminal domain of OmpA-B family members is likely extended from the membrane through the slime layer, it is possible that proper configuration of the surface lipooligosaccharides is important for proper arrangement of these surface proteins. While the slime layer is characteristic of the SFG rickettsiae, in *R. peacockii* the slime layer is thin and not always discernable [42]. The increase in the thickness of the slime layer in *R. rickettsii* upon tick feeding correlates with the restoration of virulence [43]. Genes for a sugar reductase and sugar epimerases including *CapD*, predicted to be involved in slime layer biosynthesis [44] are located very close to a mutant putative phosphoethanolamine transferase gene in *R. peacockii*. The putative phosphoethanolamine transferase gene is found between nucleotides 1176591..1178159 (Table 2) with two frameshift mutations close together that introduce a stop codon truncating the transferase domain. This enzyme is required for the correct structure of surface lipooligosaccharides of *Neisseria meningitides* and mutation of phosphoethanolamine transferase decreases bacterial binding to endothelial cells 10 fold [45], [46]. Our experience with *R. peacockii* in culture is that it binds very poorly to host cells and extracellular *R. peacockii* are observed more abundantly in vitro than other rickettsial species maintained in our laboratory [47]. The protein sequences of phosphoethanolamine transferases are not well conserved among bacteria except around the transferase domain, which is where significant homology exists to this rickettsial protein. This putative phosphoethanolamine transferase shares the same 5-transmembrane structure with others as well (Text S5). The locus tag in *R. rickettsii* SS is A1G_02570 and closely related genes are found in all other *Rickettsia* spp.

Another deletion via recombination between transposons removes A1G_04605 (*YhbC*) and A1G_04620 (transcriptional regulator of the RirA / Rrf-2 superfamily). *YhbC* was picked up in a mutant screen for virulence factors of *Salmonella enteritidis* due to its effect on the growth rate of the bacteria, making the mutant a potential live vaccine candidate [48]. The growth rate of *R. peacockii* in culture is slower than other rickettsiae grown in our lab. Transcription factors of the rirA type are repressors containing an iron-sulfur cluster, and thus can sense iron concentrations as well as nitric oxide which dissociates the cluster and alters DNA binding. The lack of iron or presence of nitric oxide leads to derepression of genes regulated by rirA, so lack of rirA protein results in an increase in expression of these regulated genes [49], [50] [51]. In the *rirA* mutant *Sinorhizobium meliloti* a toxic amount of iron builds up and leads to a hypersensitivity to H2O2 [52]. *R. peacockii* are not found in hemocytes while *R. rickettsii* are commonly found in hemocytes and this deletion of *rirA* may contribute to this observation, given that it has been shown that reactive oxygen species are produced in cattle tick hemocytes [53] and presumably in other tick species as well.

*R. peacockii* also has other deletions and mutations, notably genes that are conserved in other rickettsiae like the methyltransferase A1G_03950 (Table 2) and hypothetical gene A1G_03530 (Table 3). Nonsense mutations in *R. rickettsii* SS vs *R. peacockii* are shown in Text S6. DNA sequence found in *R. peacockii* and not in *R. rickettsii* SS is shown in Text S1 and includes mainly the ISRpe1 transposons, the three fragments of the tra cluster, the 10.5 kb fragment present in *R*. *rickettsii* Iowa vs. *R. rickettsii* SS [6] and a tandem gene duplication of A1G_02330 (RPR_04375 and RPR_04376).

Our results support the speculation that in the past, a virulent SFG rickettsia underwent changes to become the East Side Agent (*Rickettsia peacockii*) [2]. Gene reduction in rickettsiae and some other bacteria correlates with an increase in virulence [54], [55], [56] but our analysis of gene loss in *R. peacockii* suggests that transposon mediated gene reduction is responsible for avirulence in this case.

*R. peacockii* has a dynamic genome that has been and is likely still being shaped by ISRpe1 activity. The changes have resulted in a dramatic lack of synteny with *R. rickettsii* and likely contributed to rendering it non-pathogenic for vertebrates, restricting it to the tick host. *R. peacockii* has lost several genes that appear important in the transmission of pathogenic rickettsiae to a vertebrate host. At the same time it has retained a gene repertoire that enables it to survive and grow in the tick and to be transmitted transovarially to the tick's progeny. The extensive remodeling of the genome makes reversion to pathogenicity unlikely unless new virulence genes are imported. Ticks encounter and interact with many bacteria during their life cycle, some of which can invade the ovaries and cohabit the same cell (e.g. the *Francisella*-like *D. andersoni* symbiont and *C*. Cardinium spp.). We propose that symbionts such as *R. peacockii* could conceivably acquire novel genes via lateral gene transfer through their interactions with a range of bacteria including pathogens acquired by the tick during its blood meal [57]. Ticks have mechanisms for excluding foreign bacteria during the internalization of the blood meal but feeding on a heavily infected mammal may provide a challenge to this system. The acquisition of DNA from *P*. *aeruginosa* onto the *R. peacockii* plasmid may relate to the fact that ticks absorb cells and large macromolecules intracellularly for digestion [57] and *P*. *aeruginosa* is known to secrete large amounts of genomic DNA [58]. *R. peacockii* and its acquisition of mobile DNA is a good example of the ‘intracellular arena’ hypothesis at work, in that obligate intracellular bacteria more readily share genetic material if they cohabit the same cells [59]. Obligate intracellular bacteria like rickettsiae that live in arthropods which feed on mammals also increase their rate of exposure to novel gene pools [60]. We see the plasmid as the only recent recipient of foreign DNA, other than the ISRpe1 transposon, in the genome of *R. peacockii*.

## Materials and Methods

*Rickettsia peacockii* Rustic [42] was grown in *Ixodes scapularis* cell line ISE6 [47], [61] for eight in vitro passages. Genomic DNA was prepared from rickettsiae released from infected cells by forcing suspended cells five times through a 25 G needle attached to a 5 ml syringe. The resulting lysate was centrifuged at 270 rcf for 5 min to remove whole cells and the supernatant filtered through a 1.2 µm syringe filter (Whatman Puradisc FP30; Sigma-Aldrich St. Louis, MO). Rickettsiae were recovered from the filtrate by centrifugation (18,400 rcf, 5 min 4°C), resuspended in Dulbecco's Phosphate Buffered Saline (PBS) containing calcium and magnesium (Mediatech, Inc. Herndon, VA) and DNase I (15 µg/ml; from bovine pancreas Type II-S, Sigma-Aldrich), and incubated at room temperature for 30 min. After DNase I treatment to remove contaminating *Ixodes* DNA rickettsiae were centrifuged again (18,400 rcf, 5 min, 4°C) and genomic DNA was prepared using the Puregene kit (Gentra Systems, Minneapolis, MN) following the protocol for Gram negative bacteria. The *C*. Cardinium spp. isolate [13] was grown and DNA isolated in the same manner as above.

DNA was pyrosequenced on a 454FLX machine (454-Roche, Branford, CT) (226,040 reads, >30X coverage) at the BioMedical Genomics Center, U of M, St. Paul, MN and assembled using Newbler (454-Roche) requiring 99% homology; 56 contigs 500 bases or larger were obtained. To determine if transposons occupied the gaps, 50–100 basepairs from each end of the ISRpe1 transposon were used to recover 454 traces using Blastn and assembled using Sequencher (Gene Codes, Ann Arbor, MI) requiring 100% homology. These contigs were then assembled onto the ends of the original 454 generated contigs. The ISRpe1 transposons are too similar to one another to be assembled from 454 traces and mapping the contigs to the *R. rickettsii* genome followed by PCR across the gaps yielded artifactual results, again due to the similarity between individual transposons and their interaction during PCR. Therefore, contigs were extended using GenomeWalker (Clontech, Mountain View, CA) ligation mediated PCR using a single gene specific primer for each contig end. (nested PCR was unnecessary). Four GenomeWalker libraries were made using EcoRV, HaeIII, PvuII and HpaI which do not cut in the transposon. The last gap could not be filled this way, nor with standard PCR and it appeared that two transposons were present here. All contigs from the 454 assembly were then assembled onto the linear genome contig, and with the exception of contigs from *Ixodes scapularis* mitochondrial and genome sequence, only one remained unassembled. This contig contained the junction of a transposon and a transposon fragment. This junction sequence was used to retrieve 454 traces using blastn that were then assembled using Sequencher, requiring 100% homology. Primers were designed to bind to unique sequence within this junction for use in PCR with gene specific primers from the ends of the large contig. A primer was designed from a region of the transposon not found in the transposon fragment to confirm the sequence at the junction. The GenomeWalker PCR products were sequenced with transposon specific primers. AccuTaq DNA polymerase (Sigma-Aldrich) was used throughout. The coverage layout along the contigs was calculated with 454 de novo assembler software (version 2.0.00.20) using the derived file 454AlignmentInfo.tsv in 100 nucleotide scale and visually scanned for anomalies. One contig had twice the normal number of traces per unit contig length and this region was investigated with PCR and was found to contain a gene duplication (RPR_04375 and RPR_04376) that was originally assembled into one gene. The results are a circular chromosome of 1,288,492 bp and a circular plasmid of 26,406 bp.

### Annotation and Analysis

The genome was annotated using PGAAP at NCBI (http://www.ncbi.nlm.nih.gov/genomes/static/Pipeline.html). Apparent frameshifts were examined manually by recovering 454 traces from each area using Blastn and assembling them using Sequencher requiring 90% homology to determine the validity of the sequence. In ten of the areas PCR and sequencing was carried out to validate the sequence. The sequence was subjected to manual annotation by viewing the .gbf file using Artemis [9] and editing of the .sqn file. Gene fragments from apparent gene reduction auto-annotated as orfs by PGAAP were extended by blast analysis and re-annotated as misc_features (152) or removed.

Artemis Comparison Tool (ACT) (The Sanger Institute, Cambridge, UK) was used to compare the *R. peacockii* genome to that of *R. rickettsii* SS. Unique DNA sequence of each was extracted as a text file and examined using blast analysis. The level of synteny (or lack thereof) between the two genomes was examined by using Mauve [8] (Figure 1), ACT and a dotplot comparison (Figure S1). Mauve and Artemis were used to determine where the presence of a transposon coincided with a change in synteny between *R. peacockii* and *R. rickettsii* SS. Blastn and the backbone file from Mauve were used to examine all deletions in *R. peacockii* over 100 bp in size, to find which deletion junctions in *R. peacockii* were found within 5 bp of an ISRpe1 transposon (Text S2).

## Supporting Information

Figure S1 A dot plot comparison of the *R. peacockii* and *R. rickettsii* genomes.
(0.01 MB PNG)

Text S1 DNA sequence found in *R. peacockii* and not in *R. rickettsii* SS.
(0.07 MB DOC)

Text S2 Deletions in *R. peacockii* and their association with ISRpe1 transposons.
(0.26 MB DOC)

Text S3 Phylogenetic analysis of the rickettsial small hsp proteins.
(0.15 MB DOC)

Text S4 Kyte-Doolittle plots of hydrophobicity for the three small hsp/chaperone proteins from *R. peacockii*.
(0.04 MB DOC)

Text S5 Support for *R. rickettsii* A1G_02570 as phosphoethanolamine transferase.
(0.05 MB DOC)

Text S6 Nonsense mutations in *R. rickettsii* Sheila Smith relative to *R. peacockii*.
(0.04 MB DOC)

Text S7 Accession numbers for proteins in Figures 2 and 3.
(0.05 MB DOC)
