# Supplementary figures and images for: Genome Sequence of the Endosymbiont Rickettsia peacockii and Comparison with Virulent Rickettsia rickettsii: Identification of Virulence Factors
Source: PLoS One. 2009 Dec 21;4(12):e8361. doi: 10.1371/journal.pone.0008361 (PMC2791219; doi:10.1371/journal.pone.0008361)

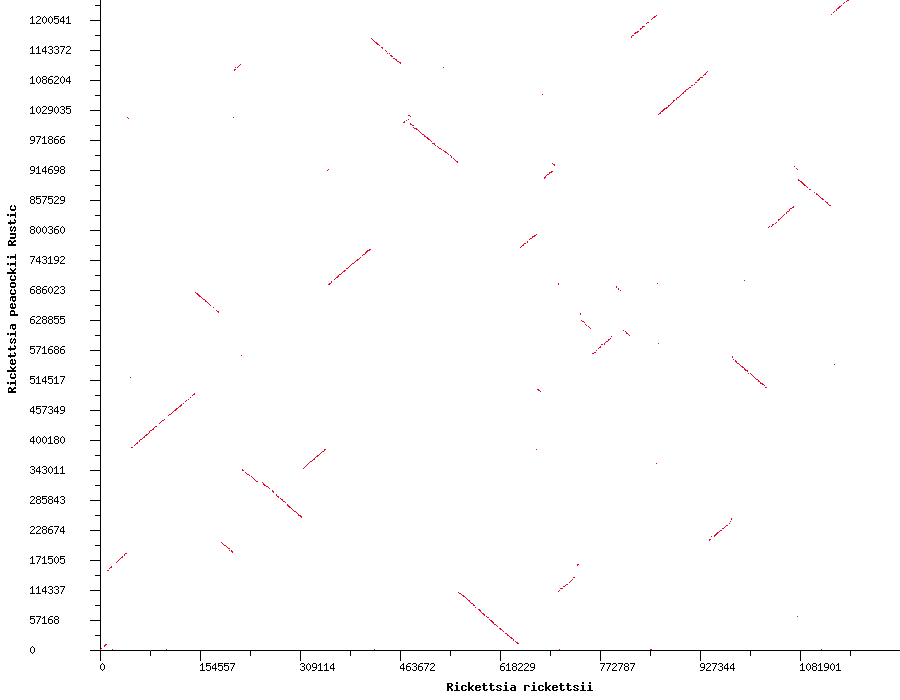

Supplement: Figure S1 — A dot plot comparison of the R. peacockii and R. rickettsii genomes. (0.01 MB PNG) [file pone.0008361.s001.png]
