# Supplementary material for: Genome Sequence of the Endosymbiont Rickettsia peacockii and Comparison with Virulent Rickettsia rickettsii: Identification of Virulence Factors
Source: PLoS One. 2009 Dec 21;4(12):e8361. doi: 10.1371/journal.pone.0008361 (PMC2791219; doi:10.1371/journal.pone.0008361)
Supplement: Text S1 — DNA sequence found in R. peacockii and not in R. rickettsii SS. (0.07 MB DOC) [file pone.0008361.s002.doc]

| **Supplemental file 1. DNA sequence found in *R. peacockii* and not in *R. rickettsii* SS** | | | | | |
| --- | --- | --- | --- | --- | --- |
|  |  | | | | |
| Locus_tag / location  in *R. peacockii* | Genes / proteins found at location | | | | |
|  | | | | |
|  |  | | | | |
| RPR_03725 | Single ankyrin repeat containing protein / likely fragment | | | | |
| RPR_00230 | Antitoxin protein | | | | |
| RPR_03885 | Membrane-associated phospholipid phosphatase, present in *R. rickettsii* Iowa. | | | | |
| RPR_03890 | LRR_RI, Leucine-rich repeats (LRRs), ribonuclease inhibitor (RI)-like subfamily, present in *R. rickettsii* Iowa. | | | | |
| RPR_04376 | Hypothetical protein with TPR repeat region. Gene duplication of RPR_04375. 744448..745607 duplicate of 745608..746767. | | | | |
| 142335..149261 | *R. bellii*-like sequence, end of tra cluster with TraV fragment, TraB, TraE leucine-rich protein. | | | | |
| 497519..499744 | *R. bellii*-like sequence, other end of the tra cluster. | | | | |
| 806589..813116 | *R. bellii*-like sequence, TraA, TraD, permease region, all with frameshift mutations. | | | | |
|  |  | | | | |
|  |  | | | | |
| **Locations of the ISRpe1 transposon:** | | | | | |
| 73768..74955 | | 496331..497519 | | 911308..912496 | |
| 84632..85820 | | 505876..507064 | | 927214..928402 | |
| 112248..113436 | | 567551..568739 | | 931028..931293 fragment | |
| 141146..142334 | | 569753..570941 | | 936546..937734 | |
| 155395..156582 | | 604046..605234 | | 942820..944008 | |
| 161153..162341 | | 607688..608876 | | 1019964..1021152 | |
| 166814..168001 | | 618761..619949 | | 1032585..1033773 | |
| 179483..180671 | | 650785..651923 | | 1036400..1037588 | |
| 210902..212090 | | 692733..693921 | | 1121688..1122876 | |
| 246264..247452 | | 704770..705958 | | 1185526..1186713 | |
| 253880..255068 | | 711554..712742 | | 1228271..1229459 | |
| 299356..300544 | | 777502..778690 | | 1229460..1230074 fragment | |
| 324335..325523 | | 805400..806588 | |  | |
| 350252..351440 | | 813117..814305 | |  | |
| 390663..391851 | | 858575..859763 | |  | |
|  | | | | | |
| Most of the other sequences found in *R. peacockii* that are not in *R. rickettsii* SS, are areas of gene reduction in other rickettsiae. Some examples, approximate locations: | | | | | |
| 6366..6584 | | | 498337..499488 | | 916577..917182 |
| 92257..93153 | | | 499527..499675 | | 926072..926293 |
| 149262..151437 | | | 563344..565294 | | 1075043..1075889 |
| 170984..171227 | | | 565317..565767 | | 1097455..1097885 |
| 249644..252817 | | | 577690..578817 | | 1097911..1098049 |
| 281904..282465 | | | 578843..579247 | | 1100650..1100839 |
| 282713..282911 | | | 621939..622580 | | 1103560..1103776 |
| 296371..296511 | | | 637714..646861 | | 1189746..1189868 |
| 414037..414590 | | | 707534..707691 | | 1235622..1235961 |
| 427368..427535 | | | 707777..707898 | | 1279566..1279802 |
| 497517..498314 | | | 769423..769622 | | 1281379..1281741 |
