# Supplementary material for: Genome Sequence of the Endosymbiont Rickettsia peacockii and Comparison with Virulent Rickettsia rickettsii: Identification of Virulence Factors
Source: PLoS One. 2009 Dec 21;4(12):e8361. doi: 10.1371/journal.pone.0008361 (PMC2791219; doi:10.1371/journal.pone.0008361)
Supplement: Text S2 — Deletions in R. peacockii and their association with ISRpe1 transposons. (0.26 MB DOC) [file pone.0008361.s003.doc]

**Supplemental file 3**. Deletions in *R. peacockii* and their association with ISRpe1 transposons.

Locations of deletions were extracted from the Mauve backbone file. All deletions greater than 100 bp were examined and the junctions located manually using blastn. The deletion was called as ISRpe1 transposon associated if a transposon was located within 5 bp of the deletion junction. Each deletion lies between the coordinates shown for *R. rickettsii* SS and the corresponding coordinates are shown for *R. peacockii*. Deletions less than 100 bp are shown if transposon associated. As an example a screen shot from Mauve is shown below the table.

|  |
| --- |
| | SS left side coordinate | SS right side coordinate | RPR coordinate of SS left side | RPR coordinate of SS right side | size of deletion | number of deletion junctions within 5 bp of transposon | deletion associated with change in synteny | | --- | --- | --- | --- | --- | --- | --- | |  |  |  |  |  |  |  | | 14498 | 15082 | 155396 | 156583 | 583 | 2 | No | | 19653 | 25701 | 161153 | 168002 | 6047 | 2 | Yes | | 36902 | 37126 | 179483 | 180672 | 223 | 2 | No | | 48049 | 52257 | 1028904 | 391853 | 4207 | 1 | Yes | | 219917 | 220001 | 1032581 | 1122876 | 83 | 2 | Yes | | 232280 | 232351 | 1135083 | 568740 | 70 | 1 | Yes | | 258009 | 266392 | 325529 | 324334 | 8382 | 2 | No | | 333287 | 333616 | 255067 | 351443 | 328 | 2 | Yes | | 372621 | 372708 | 390663 | 928402 | 86 | 2 | Yes | | 444166 | 444318 | 777501 | 1185442 | 151 | 1 | Yes | | 494539 | 498554 | 1135110 | 1021153 | 4014 | 1 | Yes | | 506267 | 506762 | 1028904 | 1036399 | 494 | 1 | Yes | | 561101 | 563976 | 968225 | 968224 | 2874 | 0 | No | | 588183 | 589234 | 944010 | 112248 | 1050 | 2 | Yes | | 614711 | 615355 | 85821 | 84633 | 643 | 2 | No | | 619349 | 619541 | 80642 | 80642 | 191 | 0 | No | | 687771 | 689062 | 151409 | 778690 | 1290 | 1 | Yes | | 691605 | 692087 | 781231 | 781235 | 481 | 0 | No | | 708170 | 708343 | 797281 | 797282 | 172 | 0 | No | | 715158 | 716455 | 804056 | 804070 | 1296 | 0 | No | | 723952 | 728058 | 499745 | 912574 | 4105 | 0 | Yes | | 730884 | 731135 | 915403 | 915404 | 250 | 0 | No | | 741167 | 741466 | 926072 | 926295 | 298 | 0 | No | | 742387 | 742859 | 927214 | 942820 | 471 | 2 | Yes | | 747947 | 753101 | 937734 | 113439 | 5153 | 2 | Yes | | 780788 | 783183 | 141145 | 162342 | 2394 | 2 | Yes | | 787638 | 788105 | 166814 | 650787 | 466 | 2 | Yes | | 807386 | 807708 | 619946 | 570941 | 321 | 2 | Yes | | 813412 | 813703 | 576671 | 576670 | 290 | 0 | No | | 839333 | 839525 | 604044 | 605235 | 191 | 2 | No | | 841975 | 846686 | 607685 | 704770 | 4710 | 2 | Yes | | 856816 | 859634 | 693922 | 618758 | 2817 | 2 | Yes | | 869412 | 871928 | 608871 | 1186713 | 2515 | 2 | Yes | | 913278 | 917419 | 1228271 | 1037588 | 4140 | 2 | Yes | | 975118 | 975936 | 1096343 | 1096344 | 817 | 0 | No | | 1000150 | 1000436 | 1121688 | 212093 | 285 | 2 | Yes | | 1139429 | 1140228 | 858574 | 936545 | 798 | 2 | Yes | | 1145752 | 1146794 | 931294 | 911307 | 1041 | 2 | Yes | |


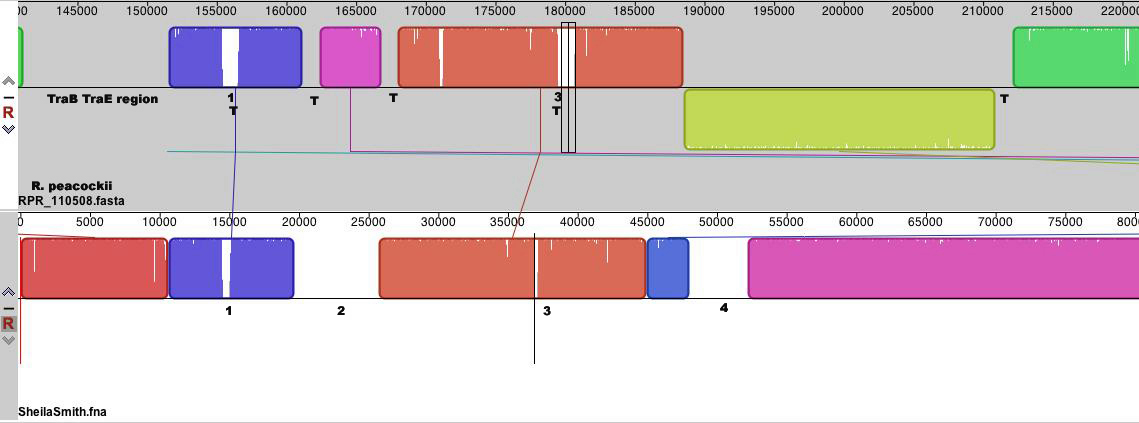


Example of deletions in R. peacockii

T = Location of ISRpe1 transposon

1 = ISRpe1 associated deletion with no effect on synteny

2 = ISRpe1 associated deletion with change of synteny, Sca1 deletion

3 = ISRpe1 associated deletion with no effect on synteny, DFR deletion

4 = ISRpe1 associated deletion with change of synteny, region of gene fragments
