# Supplementary material for: Genome Sequence of the Endosymbiont Rickettsia peacockii and Comparison with Virulent Rickettsia rickettsii: Identification of Virulence Factors
Source: PLoS One. 2009 Dec 21;4(12):e8361. doi: 10.1371/journal.pone.0008361 (PMC2791219; doi:10.1371/journal.pone.0008361)
Supplement: Text S3 — Phylogenetic analysis of the rickettsial small hsp proteins. (0.15 MB DOC) [file pone.0008361.s004.doc]

**Supplemental File 4**. Phylogenetic analysis of the rickettsial small hsp proteins. Plasmid borne copies labelled as such.


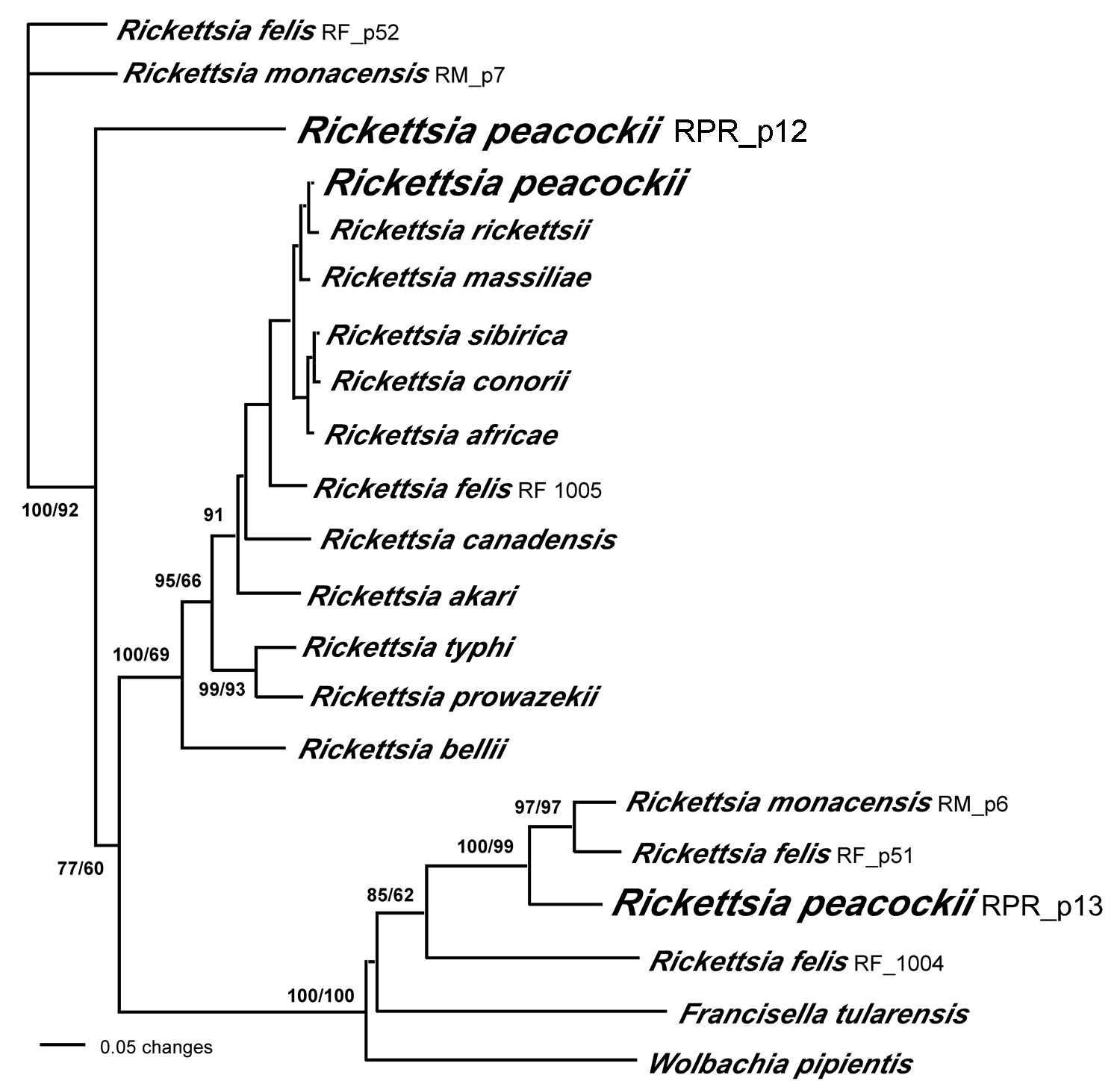


Phylogenetic analysis of rickettsial small hsp proteins. Neighbor joining (NJ) and maximum parsimony (MP) analyses included 21 taxa. Exclusion of gaps left 123 amino acids for the analyses; 9 amino acids were constant, 11 of the variable amino acids were parsimony uninformative and 103 of the variable amino acids were parsimony informative. Bootstrap analysis involved 2,000 replicates: top number is NJ bootstrap value and bottom number the MP bootstrap value.

Genbank references for the proteins used in the analysis:

>gi|238651171|ref|YP_002922008.1| small heat shock protein [Rickettsia peacockii str. Rustic]

>gi|238651172|ref|YP_002922009.1| small heat shock protein [Rickettsia peacockii str. Rustic]

>gi|238650498|ref|YP_002916350.1| small heat shock protein [Rickettsia peacockii str. Rustic]

>gi|190015793|ref|YP_001967389.1| small heat shock protein [Rickettsia monacensis]

>gi|67459845|ref|YP_247468.1| small heat shock protein [Rickettsia felis URRWXCal2]

>gi|91205652|ref|YP_538007.1| small heat shock protein [Rickettsia bellii RML369-C]

>gi|15604143|ref|NP_220658.1| HEAT shock protein (hsp22) [Rickettsia prowazekii str. Madrid E]

>gi|15892286|ref|NP_360000.1| heat shock protein [Rickettsia conorii str. Malish 7]

>gi|157964346|ref|YP_001499170.1| small heat shock protein [Rickettsia massiliae MTU5]

>gi|157803495|ref|YP_001492044.1| small heat shock protein [Rickettsia canadensis str. McKiel]

>gi|34580701|ref|ZP_00142181.1| heat shock protein [Rickettsia sibirica 246]

>gi|167471487|ref|ZP_02336191.1| heat shock protein [Rickettsia africae ESF-5]

>gi|157828241|ref|YP_001494483.1| heat shock protein [Rickettsia rickettsii str. 'Sheila Smith']

>gi|51473470|ref|YP_067227.1| HSP22-like heat shock protein [Rickettsia typhi str. Wilmington]

>gi|157825492|ref|YP_001493212.1| heat shock protein [Rickettsia akari str. Hartford]

>gi|67459397|ref|YP_247021.1| small heat shock protein [Rickettsia felis URRWXCal2]

>gi|190015792|ref|YP_001967388.1| small heat shock protein [Rickettsia monacensis]

>gi|67459844|ref|YP_247467.1| small heat shock protein [Rickettsia felis URRWXCal2]

>gi|67459396|ref|YP_247020.1| small heat shock protein [Rickettsia felis URRWXCal2]

>gi|56708782|ref|YP_170678.1| heat shock protein [Francisella tularensis subsp. tularensis SCHU S4]

>gi|190571022|ref|YP_001975380.1| heat shock protein, Hsp20 family [Wolbachia endosymbiont of Culex quinquefasciatus Pel]
