# Supplementary material for: Genome Sequence of the Endosymbiont Rickettsia peacockii and Comparison with Virulent Rickettsia rickettsii: Identification of Virulence Factors
Source: PLoS One. 2009 Dec 21;4(12):e8361. doi: 10.1371/journal.pone.0008361 (PMC2791219; doi:10.1371/journal.pone.0008361)

**Supplemental File 5**. Kyte-Doolittle plots of hydrophobicity for the three small hsp / chaperone proteins from *R. peacockii*.

RPR_2300 chromosomal gene:


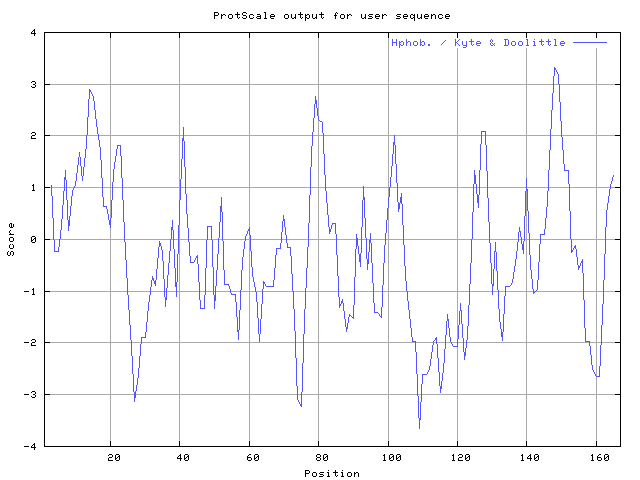


RPR_p12, on plasmid


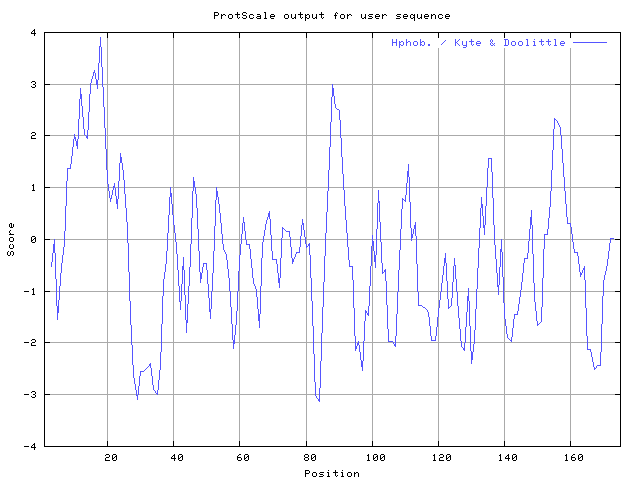


RPR_p13 on plasmid


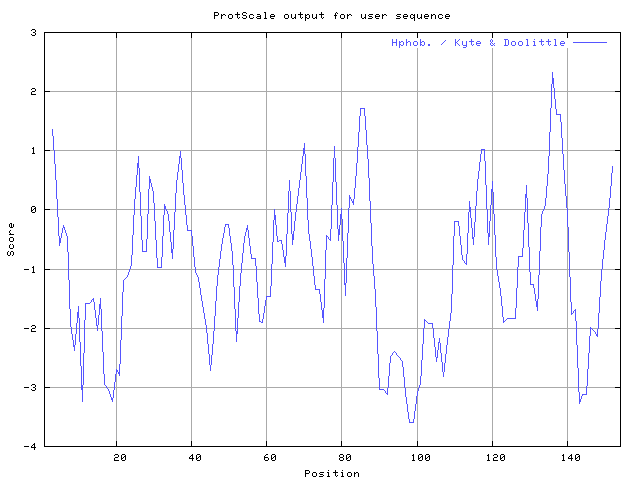

Supplement: Text S4 — Kyte-Doolittle plots of hydrophobicity for the three small hsp/chaperone proteins from R. peacockii. (0.04 MB DOC) [file pone.0008361.s005.doc]
