# Supplementary material for: Genome Sequence of the Endosymbiont Rickettsia peacockii and Comparison with Virulent Rickettsia rickettsii: Identification of Virulence Factors
Source: PLoS One. 2009 Dec 21;4(12):e8361. doi: 10.1371/journal.pone.0008361 (PMC2791219; doi:10.1371/journal.pone.0008361)
Supplement: Text S5 — Support for R. rickettsii A1G_02570 as phosphoethanolamine transferase. (0.05 MB DOC) [file pone.0008361.s006.doc]

**Supplemental File 6**.

Alignment of putative *R. peacockii* phosphoethanolamine transferase with homolog in *Neisseria meningitides* and Phobius plots of the *R. rickettsii* homolog and the top blastP hit annotated as phosphoethanolamine transferase when using the *R. rickettsii* homolog as the query.

Alignment: A) From tblastn, query is the *N*. *meningitides* protein LptA (locus tag NMB1638) referred to in the text. Subject is the *R. peacockii* chromosome sequence. Premature stop codon highlighted in red near genome coordinate 1,177,860. B) Below is a similar alignment to the top blastP hit annotated as phosphoethanolamine transferase when using the *R. rickettsii* homolog as the query.

**A. tblastn using Ent638_0178 as query**

Score = 184 bits (468), Expect = 5e-48, Method: Compositional matrix adjust.

Identities = 151/542 (27%), Positives = 247/542 (45%), Gaps = 59/542 (10%)

Frame = +3

Query 11 KLSFLLALYIGLFMNGAVFFRRFDGYAQDFTVWKGVSAVVELVGTVLVTFFLLRMLSLFG 70

KLS +LA L N A+ +FD Y T+++G+ +EL ++ ++ FG

Sbjct 1176633 KLSAILAFIYCLLFNTAILIYKFDYYKA--TIFRGI---LEL-SKDFCYIYIFSFIAFFG 1176794

Query 71 RRMWRVLASLVVVF----SAGASYYMTFMNVVIGYGIIASVMTTDIDLSKEVVGLHFILW 126

+ R++ + V F SA ASYY+ F + +I S +TD++ E+ + I+W

Sbjct 1176795 LSVHRLVLKIGVGFLFITSAIASYYIYFFKINPTKQVIGSFFSTDLNEVYELTSIKLIIW 1176974

Query 127 LVCVSALPLLFIWSNRCRYTLLRQMRTPGQRIRSVTLVVLAGLMVWGPIRLLDVKQKYDE 186

++ LL C Y +L+ + L+ A L+++

Sbjct 1176975 II----FCLL-----TCFY-ILKSFAAENSKSFVTKLLSTACLLIF-------------- 1177082

Query 187 RTSGVDMPSYGGVVANSYLPSNWISALGLYAWAQVDESADNKSLKNPAKQFTYQAPKDID 246

+ PS+ + +Y P ++ L E N + + +KQ+ + D

Sbjct 1177083 -VYNIITPSFK--ILKNYFPIQYLHNSYLNFAGNFGE--KNYACIDISKQYNFIDKSD-K 1177244

Query 247 DTYFVFIIGETTRWDHMGILGYDRDTTPKLSQEKNLIAYRGYSCDTATKLSLRCMFVREG 306

D V +IGE+ R+DH GI GY+RDTTP L +NLI+++ S T LS+ + R

Sbjct 1177245 DIIGVLVIGESARFDHFGINGYERDTTPYLKTTQNLISFKAKSSSNLTYLSVPSLLSRYP 1177424

Query 307 GADDNPQRTLKEQNVFSVLRQLGFTSDLYAMQSEMWFYSNTMAQNIAYREQIGAEPRNRG 366

+ R +E + S+L LGF + Q+ M ++N NI P

Sbjct 1177425 ASQIENSR--QENSFLSILTNLGFNTTWIGTQTLMRSFANFDLSNIYNDVNFTIVPGGSA 1177598

Query 367 K-SVDDM--LLIDEMKNSLNGNPDGKHMIILHTKGSHFNYTQRYPRNFAKWTPEC----V 419

S++D ++ +K L + K +++HT GSH+NY RYP+ F TP C

Sbjct 1177599 LFSLNDHDEKILPFIKEILTNSE--KQFLVVHTSGSHWNYNARYPKEFEYCTPTCPIKVK 1177772

Query 420 GVDKDCTKDELVNSFDNSVMYVDHFIDSVIDQVRDKKAIVFYAADHGESINEFEHL-HGT 478

G DC K LVNS+DNS++Y D F ++ K +Y +DHGES+ E + HG

Sbjct 1177773 GDASDCDKLALVNSYDNSILYTDFFYII**ICLKIKMHFYYYVSDHGESLGENGYYGHGG 1177952

Query 479 PRKMAPPEQFRVPMMVWMSDKYLENPDKAKMFAQLKKEADMKVPRRHVELYDTIMGCLGY 538

P EQ VP++VW+SD + K + +K A+ ++ +V + +I+ CL

Sbjct 1177953 P---LLAEQITVPLIVWVSDDF--QAKYPKSVSSIKNYANTEISHDYV--FHSILNCLNI 1178111

Query 539 TS 540

S

Sbjct 1178112 ES 1178117

B. R. rickettsii A1G_02570 used as query

gb|EEH95397.1| phosphoethanolamine transferase [Citrobacter sp. 30_2]

Length=564

Score = 198 bits (503), Expect = 1e-48, Method: Compositional matrix adjust.

Identities = 151/539 (28%), Positives = 253/539 (46%), Gaps = 52/539 (9%)

Query 15 KLSAILAFIYCLFFNTAILIYKFDYYKA--TIFRGILELSKDFCYIYIFSFIAFFGLSVH 72

KLS +LA LF N A+ +F Y T+++G+ + + + + F+

Sbjct 11 KLSFLLAIYIGLFMNCAVFFRRFSGYAHEFTVWKGLSAVVELAGTVLVTFFLLRLLSLFG 70

Query 73 RLVLKIGVGFLFITSAIASYYIYFFKINPTKQVIGSFFSTDLNEVYELTSIKLIIWIIFC 132

R ++ F+ + SA ASYY+ F + +I S +TD++ E+ I+W++

Sbjct 71 RRAWRVLATFVVLCSAGASYYMTFLNVVIGYGIIASVMTTDIDLSKEVVGWHFIVWLVSV 130

Query 133 LL---------TCFYTL-KSFAAENSKSFVTKLLSTACLLIFV---------------YN 167

+ C YTL K + ++ A L++++

Sbjct 131 SIIPLVLIWSNRCRYTLMKQIRTPGMRWRSVAVVLLAGLMVWLPIRLLSMQQRSVERATG 190

Query 168 IITPSFK--ILKNYFPIQYLHNSYLNFAGNFGEK--NYACIDISKQYNFI-DKSDKDIIG 222

I PS+ + +Y P +L L E N + ++ +K++ ++ K+ D

Sbjct 191 IDLPSYGGVVANSYLPSNWLSALGLYAWAQVDESSDNKSLMNPAKKFTYVAPKNLDDTYV 250

Query 223 VLVIGESARFDHFGINGYERDTTPYLKTTQNLISFKAKSSSNLTYLSVPSLLSRYPASQI 282

V +IGE+ R+DH GI GYER+TTP L +NL +F+ S T LS+ + R ++

Sbjct 251 VFIIGETTRWDHMGIFGYERNTTPRLAQEKNLAAFRGYSCDTATKLSLRCMFVREGGAEE 310

Query 283 ENSR--QENSFLSILTNLGFNTTWIGTQTLMRSFANFDLSNIYNDVNFTIVPGGSALFSL 340

R +E + S+L LGF T Q+ M ++N NI P

Sbjct 311 NPQRTLKEQNVFSVLGQLGFKTDLYAMQSEMWFYSNTMAENIAYREQIGAEPRNRGK--- 367

Query 341 NDHDEKILPFIKEILTNSE---KQFLVVHTSGSHWNYNARYPKEFEYFTPTCPIKVKGDA 397

N D ++ +++ L N+E K +++HT GSH+NY RY +E+ + P C V D

Sbjct 368 NVDDMLLITEMQQSLQNNEDGDKHLIILHTKGSHFNYTQRYTREYAQWKPEC---VNVD- 423

Query 398 SDCDKLALVNSYDNSILYTDFFLYNLIDLLKDKNAFLLYVSDHGESLGENGYYGHGGP-- 455

S C K ++NS+DNS+ Y D F+ + D L+DK A + Y +DHGES+ E + HG P

Sbjct 424 SGCSKAEMINSFDNSVTYVDHFITKVFDQLRDKKAIVFYAADHGESINEREHL-HGTPRK 482

Query 456 -LLAEQITVPLIVWVSDDF--QAKYPKSVSSIKNYANTEISHDYV--FHSILNCLNIES 509

EQ VP++VW+SD + + K+ + +K A+ ++ +V + +IL CL S

Sbjct 483 MAPPEQFRVPMMVWMSDKYLEDPDHAKAFAHLKQEADMKVPRRHVELYDTILGCLGYTS 541

**Phobius plots:**

phosphoethanolamine transferase Ent638_0178

*R. rickettsii* putative phosphoethanolamine transferase,
