# Supplementary material for: Genome Sequence of the Endosymbiont Rickettsia peacockii and Comparison with Virulent Rickettsia rickettsii: Identification of Virulence Factors
Source: PLoS One. 2009 Dec 21;4(12):e8361. doi: 10.1371/journal.pone.0008361 (PMC2791219; doi:10.1371/journal.pone.0008361)
Supplement: Text S6 — Nonsense mutations in R. rickettsii Sheila Smith relative to R. peacockii. (0.04 MB DOC) [file pone.0008361.s007.doc]

| **Supplemental file 7. Nonsense mutations in *R. rickettsii* Sheila Smith relative to *R. peacockii*** | |
| --- | --- |
|  |  |
| Location in *R. rickettsii* SS | Gene product and type of mutation |
|  |  |
| 9627..10195 | COG1752 Predicted esterase of the alpha-beta hydrolase superfamily, frameshift in homolog of RPR_00050 |
| 162582..163104 | Hypothetical protein, frameshift in homolog of RPR_04060 |
| 324844..325172 | COG2154 Pterin-4a-carbinolamine dehydratase, frameshifts in homolog of RPR_01640 |
| 326526..327883 | Response regulator PleD, frameshift in homolog of RPR_01620 |
| 351032..352168 | Penicillin-binding protein 4*, frameshifts in homolog of RPR_02205 |
| 552767..552355 | Cytidyltransferase-related, putative glycerol-3-phosphate cytidyltransferase TagD, frameshift in homolog of RPR_5745 |
| 591460..591845 | COG5611, nucleic acid binding protein, contains PIN domain, frameshift in homolog of RPR_00650 |
| 623744..624260 | Gamma carbonic anhydrase-like superfamily, frameshift in homolog of RPR_00450 |
| 659733..660284 | Peptide deformylase, internal stop codon in homolog of RPR_00235 |
| 720621..722328 | 30S ribosomal protein S1, frameshift, possible sequencing error |
| 759329..760222 | Hypothetical gene, frameshifts in homolog of RPR_00685 |
| 983885..984270 | Integral membrane protein COG5528, multiply mutated homolog of RPR_06605 |
| 1022705..1023285 | Hypothetical gene, frameshift in homolog of RPR_01425 |
| 1032790.. 1033333 | Hypothetical gene, frameshift in homolog of RPR_1490 |
| 1184724.. 1185189 | Phosphatidylethanolamine-binding protein PEBP, frameshift in homolog of RPR_05060 |
