# Supplementary material for: Genome Sequence of the Endosymbiont Rickettsia peacockii and Comparison with Virulent Rickettsia rickettsii: Identification of Virulence Factors
Source: PLoS One. 2009 Dec 21;4(12):e8361. doi: 10.1371/journal.pone.0008361 (PMC2791219; doi:10.1371/journal.pone.0008361)
Supplement: Text S7 — Accession numbers for proteins in Figures 2 and 3. (0.05 MB DOC) [file pone.0008361.s008.doc]

Genbank references for the proteins used in the analysis in figure 3:

| Label on parA tree figure 3 | Genbank information |
| --- | --- |
|  |  |
| >Rickettsia peacockii pRPR | >gi|238651160|ref|YP_002921997.1| plasmid stability protein ParA [Rickettsia peacockii str. Rustic] |
| >Rickettsia felis pRF | >gi|67459816|ref|YP_247439.1| plasmid stability protein ParA [Rickettsia felis URRWXCal2] |
| >Rickettsia felis pRFδ | >gi|67459865|ref|YP_247487.1| chromosome partitioning ParA family protein [Rickettsia felis URRWXCal2] |
| >Actinobacillus porcitonsillarum pIMD50 | >gi|66968654|ref|YP_245407.1| ParA-like [Actinobacillus porcitonsillarum] |
| >Escherichia coli pB171 | >gi|10955419|ref|NP_053131.1| hypothetical protein pB171_069 [Escherichia coli] |
| >Pseudomonas syringae pPSR1 | >gi|38257043|ref|NP_940697.1| stability protein [Pseudomonas syringae pv. syringae] |
| >Rickettsia massiliae pRMA | >gi|157954018|ref|YP_001497196.1| plasmid stability protein ParA [Rickettsia massiliae MTU5] |
| >Trichoplax adhaerens | >gi|196017298|ref|XP_002118475.1| hypothetical protein TRIADDRAFT_62510 [Trichoplax adhaerens] |
| >Chlorobium limicola pCL1 | >gi|10956076|ref|NP_052163.1| hypothetical protein pCL1_p4 [Chlorobium limicola] |
| > Lawsonia intracellularis plasmid 1 | >gi|94972372|ref|YP_595592.1| chromosome-partitioning ATPase [Lawsonia intracellularis PHE/MN1-00] |
| >Xanthomonas axonopodis pAG1 | >gi|190410578|ref|YP_001965992.1| plasmid partitioning protein-like [Xanthomonas axonopodis pv. glycines] |
| >Rickettsia monacensis pRM | >gi|190015802|ref|YP_001967398.1| ParA-like protein [Rickettsia monacensis] |
| > Prosthecochloris aestuarii | >gi|194311672|gb|ACF46067.1| Cobyrinic acid ac-diamide synthase [Prosthecochloris aestuarii DSM 271] |
| > Bacteroides fragilis | >gi|52216983|dbj|BAD49576.1| chromosome-partitioning ATPase [Bacteroides fragilis YCH46] |
| >Rickettsia africae pRAF | >gi|228478462|ref|YP_002845772.1| Plasmid stability protein ParA [Rickettsia africae ESF-5] |
| > Shewanella oneidensis megaplasmid | gi|112949654|gb|AAN52945.2| ParA family protein [Shewanella oneidensis MR-1] |
| > Vibrio shilonii | >gi|148835191|gb|EDL52166.1| ParA family protein [Vibrio shilonii AK1] |
| > Candidatus Hamiltonella defensa | >gi|229466933|gb|ACQ68707.1| plasmid partition protein ParA-like protein [Candidatus Hamiltonella defensa 5AT (Acyrthosiphon pisum)] |
| > Laribacter hongkongensis pHLHK8 | >gi|57790538|ref|YP_184745.1| ATPase [Laribacter hongkongensis] |
| > Nitrosospira multiformis plasmid 3 | >gi|82703932|ref|YP_413495.1| plasmid partition protein ParA-like [Nitrosospira multiformis ATCC 25196] |
| >Bordetella bronchiseptica pKBB4037 | >gi|85815544|emb|CAI47014.1| partition protein [Bordetella bronchiseptica] |
| >Rickettsia endosymbiont of Ixodes scapularis pREIS1 | >gi|239920842|gb|EER20867.1| ParA1 [Rickettsia endosymbiont of Ixodes scapularis] |
| >Rickettsia endosymbiont of Ixodes scapularis pREIS2 | >gi|239920783|gb|EER20809.1| chromosome partitioning ParA family protein [Rickettsia endosymbiont of Ixodes scapularis] |
| >Rickettsia endosymbiont of Ixodes scapularis pREIS3 | >gi|239920721|gb|EER20748.1| chromosome partitioning ParA family protein [Rickettsia endosymbiont of Ixodes scapularis] |
| >Rickettsia endosymbiont of Ixodes scapularis pREIS-NZGG688316.1 | >gi|239946416|ref|NZ_GG688316.1| Rickettsia endosymbiont of Ixodes scapularis plasmid scaffold scf_1118437112405, whole genome shotgun sequence,  translation of nucleotides 10818 – 11573. |
| >Rickettsia peacockii chromosomal parA | >gi|238624623|gb|ACR47329.1| chromosome partitioning protein [Rickettsia peacockii str. Rustic] |
| >Rickettsia bellii chromosomal parA | >gi|91206165|ref|YP_538520.1| ATPase [Rickettsia bellii RML369-C] |

Genbank references for the proteins used in the analysis for Figure 2:

>gi|238624491|gb|ACR47197.1| transposase ISRpe1 [Rickettsia peacockii str. Rustic]

>gi|157964455|ref|YP_001499279.1| transposase [Rickettsia massiliae MTU5]

>gi|189502309|ref|YP_001958026.1| hypothetical protein Aasi_0934 [Candidatus Amoebophilus asiaticus 5a2]

>gi|124004342|ref|ZP_01689188.1| transposase [Microscilla marina ATCC 23134]

>gi|58698163|ref|ZP_00373085.1| transposase, putative [Wolbachia endosymbiont of Drosophila ananassae]

>gi|167842390|ref|ZP_02469074.1| putative transposase [Burkholderia thailandensis MSMB43]

>gi|162148593|ref|YP_001603054.1| putative transposase [Gluconacetobacter diazotrophicus PAl 5]

>gi|27376966|ref|NP_768495.1| putative transposase [Bradyrhizobium japonicum USDA 110]

>gi|53729012|ref|ZP_00134264.2| COG2801: Transposase [Actinobacillus pleuropneumoniae serovar 1 str. 4074]

>gi|163741687|ref|ZP_02149077.1| hypothetical protein RG210_03940 [Phaeobacter gallaeciensis 2.10]

>gi|54294954|ref|YP_127369.1| hypothetical protein lpl2033 [Legionella pneumophila str. Lens]

>gi|146295020|ref|YP_001185444.1| integrase catalytic subunit [Shewanella putrefaciens CN-32]

>gi|193787996|dbj|BAG50505.1| ISSod13, transposase [Vibrio parahaemolyticus]

Cardinium ISRpe1 PCR product: GU166817
